# Supplementary material for: Responsive Microgels through RAFT-HDA Dynamic Covalent Bonding Chemistry
Source: Molecules. 2024 Mar 8;29(6):1217. doi: 10.3390/molecules29061217 (PMC10975865; doi:10.3390/molecules29061217)
Supplement: Supplementary file 1 [file molecules-29-01217-s001.zip › molecules-2801354-supplementary.pdf]

Supporting Information

Responsive Microgels through RAFT-HDA Dynamic Covalent Bonding Chemistry

Jingkai Nie [1], Hang Yin [1,*], Ruyue Cao [1], Changyuan Huang [2], Xiang Luo [2] and Jun Ji [1]

1 State Grid Smart Grid Research Institute Co., Ltd., Beijing 102211, China; niejingkai@geiri.sgcc.com.cn (J.N.); caoruyue@geiri.sgcc.com.cn (R.C.); jijun@geiri.sgcc.com.cn (J.J.)
2 China Electric Power Research Institute Co., Ltd., Beijing 100192, China; huangchangyuan@epri.sgcc.com.cn (C.H.); luoxiang@epri.sgcc.com.cn (X.L.)
* Correspondence: yinhang@geiri.sgcc.com.cn; Tel.: +86-66601587

Keywords: ultrasound-responsive microgels; phosphoryl disulfide; furan groups; hetero Diels–Alder (HDA) reaction; dynamic covalent bonding

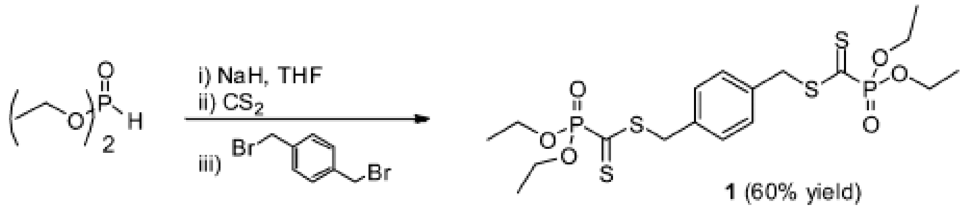

Scheme S1. Synthetic routes of (a) 1,4-phenylenebis (methylene) bis((diethoxyphosphoryl)methanedithioformate) (P-Di-linker).

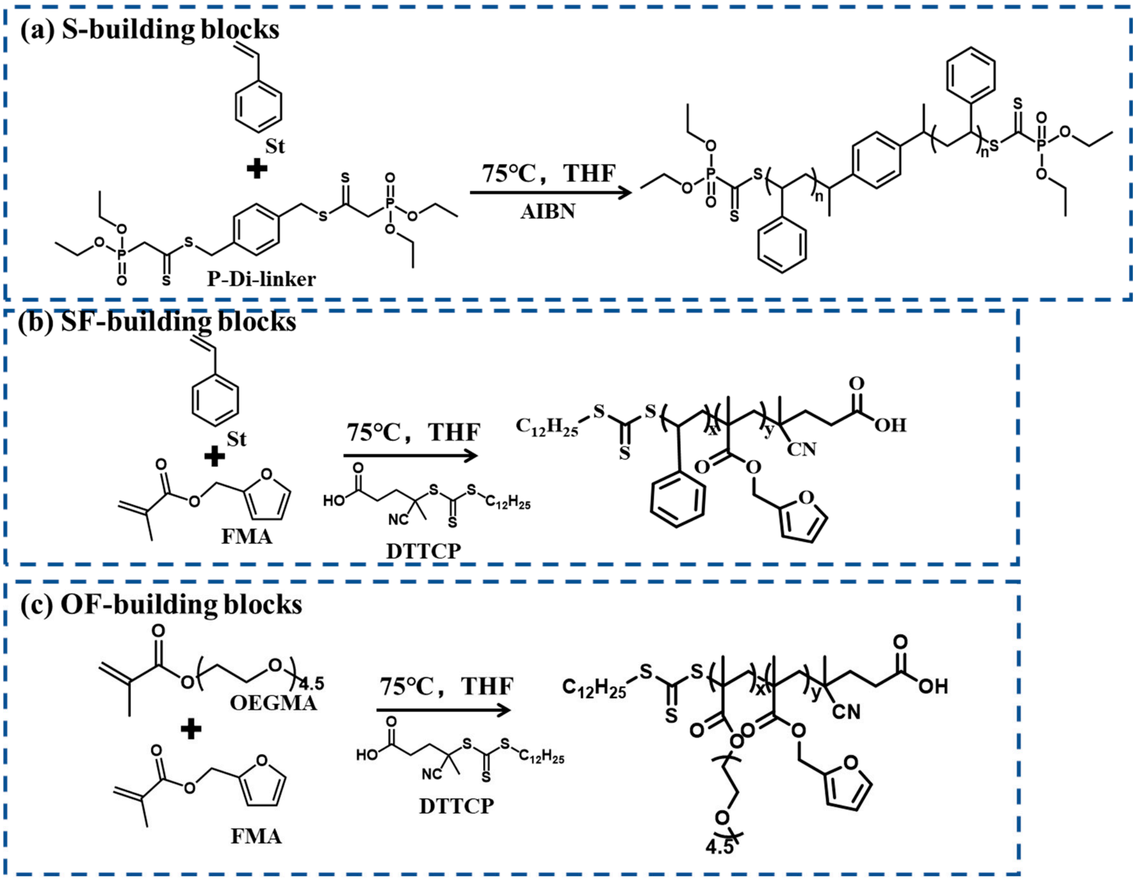

Scheme S2. (a-c) Synthetic routes of building blocks.

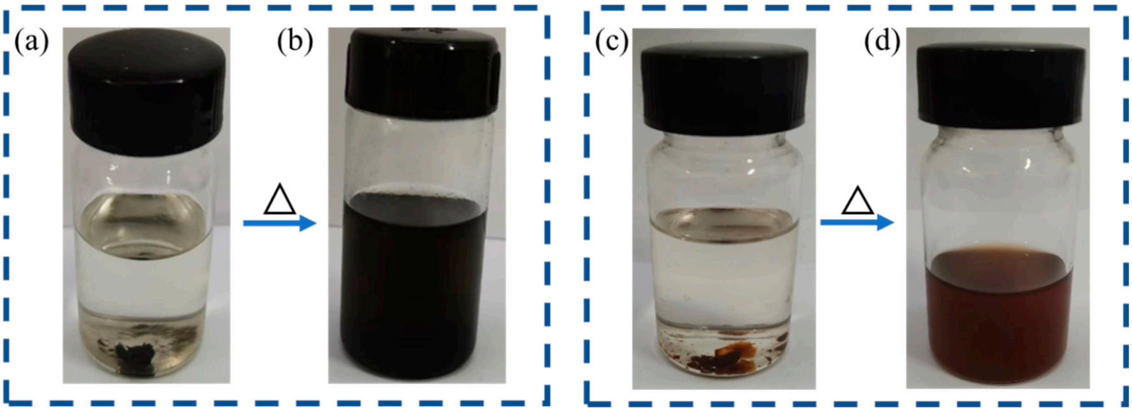

Figure S1. (a) DA product soaked in toluene at room temperature for 5 days. (b) DA product heated in toluene 120℃ for 2h. (c) DA product soaked in THF at room temperature for 5 days. (d) DA product heated in toluene 120℃ for 8h.

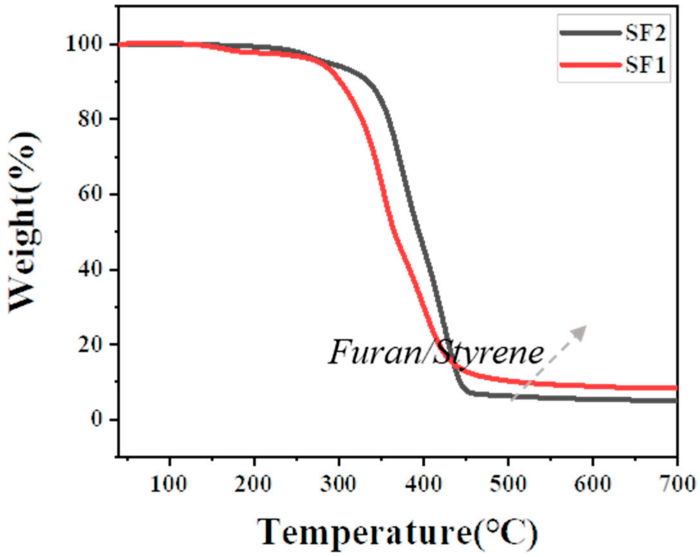

Figure S2. Comparative thermal weight loss spectra of SF1 and SF2 systems.

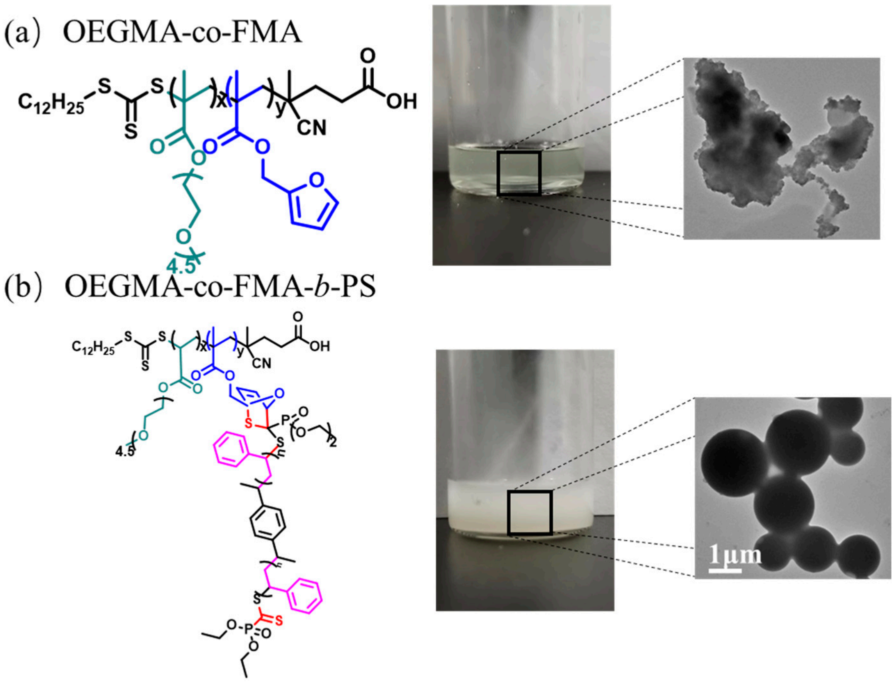

Figure S3. (a) OEGMA and FMA copolymer. (b) Furan with dithioester HDA to obtain OEGMA-co-FMA-b-PS.

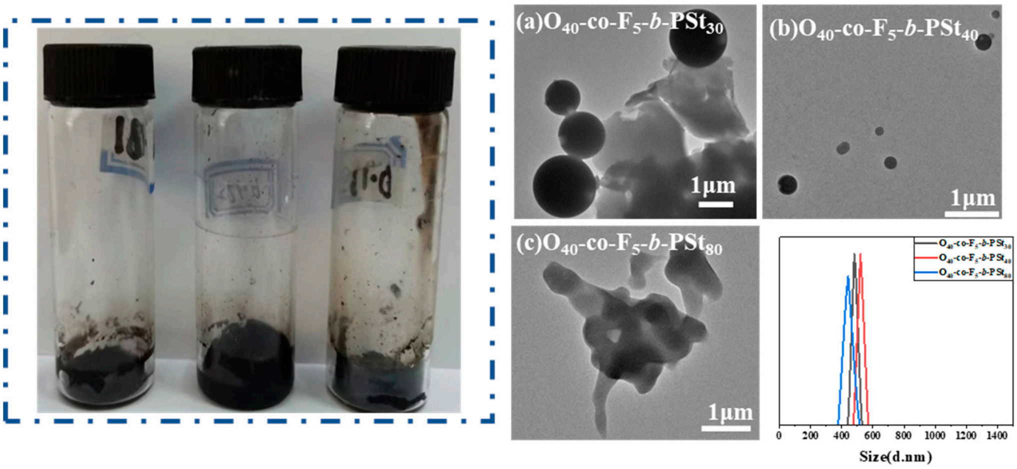

Figure S4. Photo, TEM images and DLS curves of the large aggregated system OF4-S2, OF4-S3, OF4-S4.

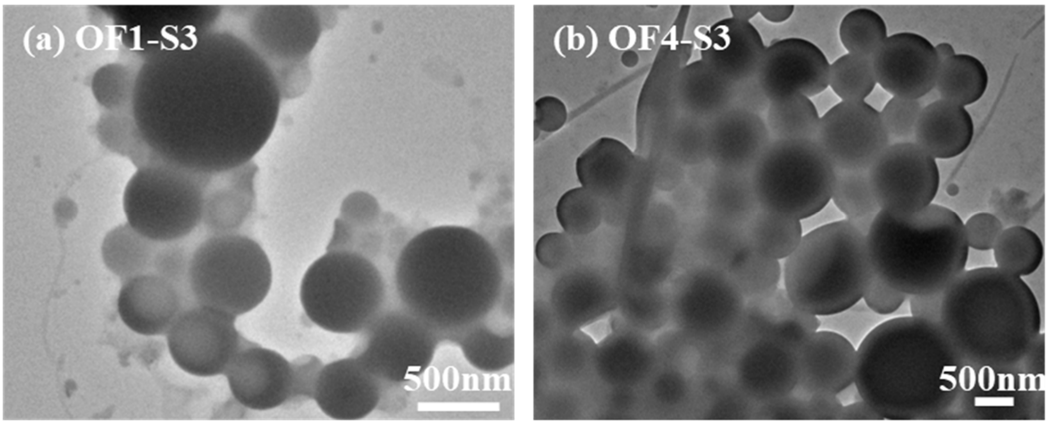

Figure S5. TEM images of microgels after 36 days at room temperature.
